# A case report of a rare etiology of an abdominal cystic lesion in adults: Peritoneal inclusion cysts

**DOI:** 10.1016/j.ijscr.2024.110792

**Published:** 2024-12-26

**Authors:** Ahmed Hadj Taieb, Mohamed Ali Chaouch, Mohamed Zayati, Ramzi Beltaifa, Besma Gafsi, Faouzi Noomen

**Affiliations:** a Department of Visceral and Digestive Surgery, Monastir University Hospital, Monastir, Tunisia; b Department of Anesthesia, Monastir University Hospital, Monastir, Tunisia

**Keywords:** Peritoneal inclusion cyst, Abdominal cystic lesion, Mesothelial cyst, Rare case, Male patient, Case report

## Abstract

**Introduction and importance:**

Peritoneal inclusion cysts (PICs), also known as peritoneal mesothelial cysts, are rare, benign cystic lesions primarily occurring in the abdominopelvic cavity of premenopausal women with histories of pelvic surgery or inflammation. These cysts can present with nonspecific symptoms and may mimic other abdominal pathologies, making diagnosis challenging.

**Case presentation:**

A 41-year-old male with no significant medical history, who experienced progressive nonspecific abdominal pain over several months. Clinical examination revealed a poorly defined mass in the right hemi-abdomen. Imaging studies, including CT and MRI, identified an elongated subhepatic cystic lesion, suggesting a peritoneal inclusion cyst or mesenteric cyst. Surgical intervention was decided following multidisciplinary team consultation. Intraoperative findings revealed a 13 cm intraperitoneal cyst with clear fluid content in the right hemi-abdomen, adherent to adjacent structures. Monobloc cystectomy was performed, and a histopathological examination confirmed the diagnosis of a peritoneal inclusion cyst.

**Clinical discussion:**

PICs are rare and typically affect females, with only limited cases reported in males. These cysts can be asymptomatic or present with non-specific symptoms such as abdominal pain or distension, often requiring imaging and, occasionally, surgical exploration for diagnosis. The differential diagnosis includes other cystic abdominal lesions, emphasizing the need for thorough clinical and imaging assessment. The preferred management of PICs is surgical excision, as it provides histological confirmation and minimizes recurrence risk.

**Conclusion:**

This case highlights a rare presentation of a PIC in a male patient, underscoring the importance of considering PICs within the differential diagnosis of abdominal cystic lesions regardless of patient gender. Surgical resection remains the primary management approach, but further research is needed to establish standardized guidelines for diagnosis and treatment.

## Highlights

• This case report documents an uncommon presentation of a peritoneal inclusion cyst in a 41-year-old male, as these cysts typically affect premenopausal women.
• Peritoneal inclusion cysts can mimic other abdominal pathologies, underscoring the importance of thorough imaging and differential diagnosis to avoid misdiagnosis.
• Surgical excision was successfully used for diagnosis and treatment, highlighting it as the preferred approach to reduce recurrence and confirm pathology.

## 1 Introduction

Peritoneal inclusion cysts (PICs), also referred to as peritoneal mesothelial cysts, are rare, benign lesions occurring primarily within the abdominopelvic cavity [1,2]. They are most commonly seen in premenopausal women with a history of pelvic surgery or inflammatory conditions such as endometriosis. Although benign, PICs can often mimic various other abdominal pathologies, including ovarian cysts, mesenteric cysts, and lymphangiomas, which can complicate both their diagnosis and management. Due to their rarity and non-specific presentation, PICs are frequently challenging to diagnose, requiring careful differentiation from other abdominal masses. Typically, PICs are asymptomatic but may present with symptoms such as abdominal pain, distention, or the presence of a palpable mass. Imaging modalities, such as ultrasound, CT, and MRI, are essential in evaluating these cysts, offering valuable insights into their size, location, and structural characteristics [3]. However, these diagnostic tools alone may not definitively distinguish PICs from other lesions, especially when atypical features are present. Adhering to SCARE guidelines [4], this case report aimed to highlight a rare etiology of an abdominal mass in a young patient.

## 2 Case presentation

**Fig. 1:**
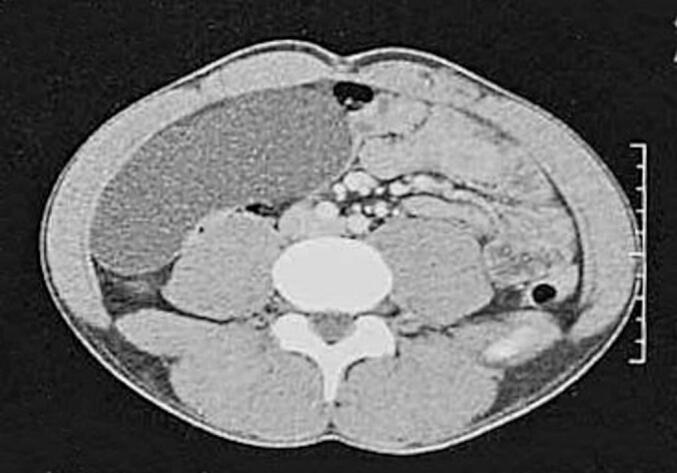
CT scan images of the peritoneal cyst.

**Fig. 2:**
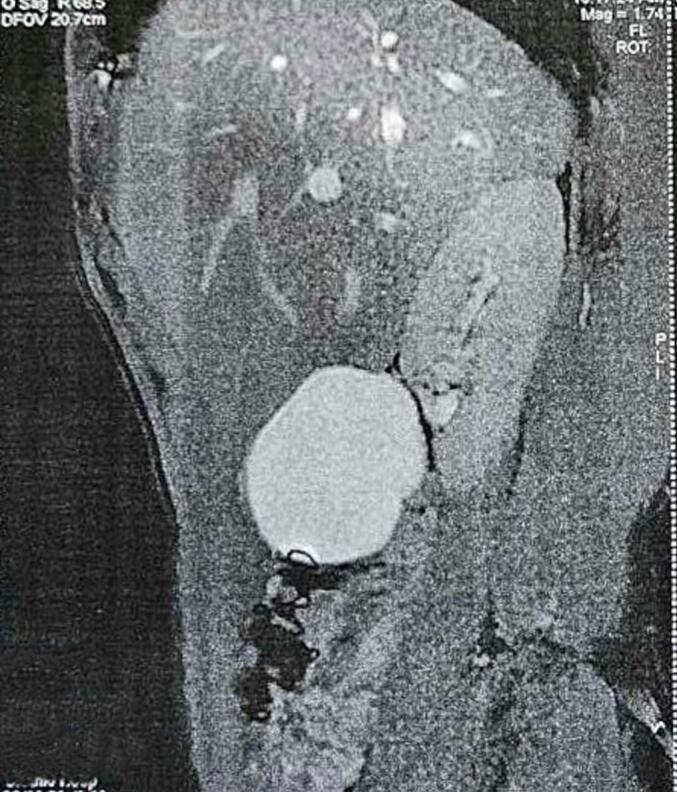
MRI images of the peritoneal cyst.

**Fig. 3:**
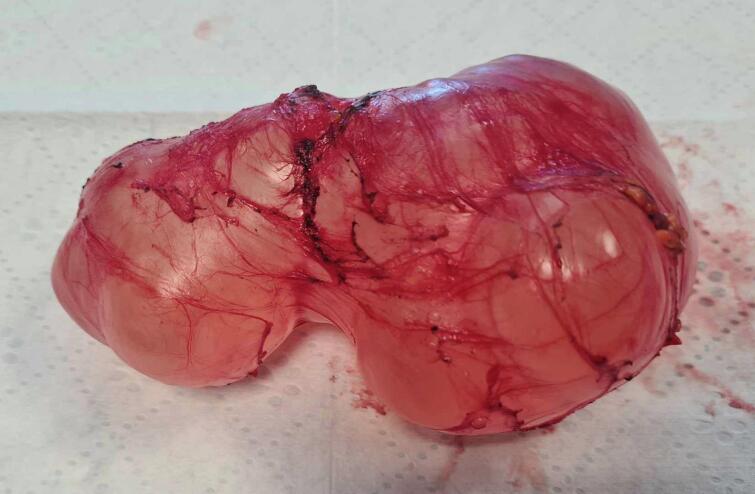
Specimen after monobloc cystectomy.

Mr. KR, a 41-year-old patient with no notable medical history, presented with nonspecific abdominal pain that had been progressing over several months, without bowel transit disturbances or general health impairment. On clinical examination, a poorly defined mass was palpated in the right hemi-abdomen; otherwise, the examination was unremarkable. The patient underwent an abdominal CT scan, which revealed an elongated subhepatic cyst with an intraperitoneal appearance, suggesting a mesenteric cyst or a cystic lymphangioma (Fig. 1). An abdominal MRI—preferred for assessing peritoneal inclusion cysts—was performed. It showed a well-defined unilocular intraperitoneal cystic lesion in the right flank, measuring 11.5 cm in its longest axis, with fluid content and no atypical features (Fig. 2). A peritoneal origin was primarily considered, suggesting a peritoneal inclusion cyst. The different imaging features showed no signs of invasion to organs such as the intestine or omentum. Following the presentation of the case at a multidisciplinary team meeting, surgical treatment was selected. Intraoperatively, a cystic mass of 13 cm in length with clear fluid content was found, located intraperitoneally in the right hemi-abdomen. This mass adhered to the right colic angle superiorly, the right colon laterally, the right kidney posteriorly, and the mesocolon, coming into contact medially with intestinal loops. A monobloc cystectomy was performed after releasing the cyst while preserving its wall intact (Fig. 3) confirming intraoperatively the non-invasion or adjacent organs in the imaging features. The postoperative course was uneventful. The Pathological examination confirmed the diagnosis of a peritoneal inclusion cyst. There was no recurrence after one year of the follow-up.

## 3 Discussion

PICs were first described by Plaut in 1928 who incidentally observed ‘loose cysts of the pelvis’ during an operation for uterine leiomyoma [1,2]. However, their mesothelial nature was confirmed later in 1979 by Menemeyer and Smith [5]. They are extremely rare. They account for 3–5 % of peritoneal mesothelial lesions, however, are benign with a low rate of transformation into malignancy [6,7]. Those lesions occur more frequently in females (80–90 %) and are most common in the third to fourth decades [7]. Although the etiology behind PICs is still unclear, they are thought to result from benign inflammatory proliferation due to various risk factors, such as prior abdominopelvic surgeries, gastrointestinal inflammation, or pelvic inflammation [6]. Still, many patients are completely asymptomatic, and up to 10 % of PICs are discovered incidentally at the time of imaging or surgery [8]. Their clinical presentation is unspecific: it is usually abdominal pain, increased abdominal girth, and constipation. Physical examination revealed abdominal distension, abdominal tenderness, or a palpable mass [3]. They can cause symptoms due to compression of nearby structures, stretching of the mesentery as a result of the cyst's rapid growth, and rupture and infection [9]. Diagnosis of PICs is based on visible methods. The ultrasound image of PICs typically is called a “spider in the web” pattern [3]. CT scan and/or MRI are useful to assess the size, characteristics, and surrounding tissue involvement in a preoperative setting [10]. Park et al. aimed to present the CT characteristics that may help to distinguish benign from malignant mesotheliomas. CT features of PIC describe a multilocular cystic mass, multiple thin-walled cysts, or a unilocular cystic mass, while the CT findings for malignant peritoneal mesotheliomas can range from a “dry” aspect, a localized form with peritoneum-based masses, to a “wet” aspect, with diffuse irregular thickening of the peritoneum, ascites and a mass involving the omentum [11]. The differential diagnosis for this illness includes hydatid cysts, lymphangiomas, ovaries-related cysts, peritoneal cysts, and cystic teratomas, which can be differentiated by thorough history-taking, physical examination, and radiological investigations [9]. Various treatment options are offered to treat peritoneal inclusion cysts. Such different modalities of treatment include but are not limited to observation, hormonal management, image-guided aspiration, image-guided sclerotherapy, potassium-titanyl-phosphate laser ablation, and surgical excision [7]. However, laparoscopic surgery is the preferred method because it offers less blood loss and a shorter hospital stay [12], with a low recurrence rate (<1 %) [13].

## 4 Conclusion

In conclusion, peritoneal inclusion cysts remain a rare pathology with limited standardized management guidelines, requiring each case to be individually assessed. Surgical resection is currently the primary management approach, allowing for histological confirmation and the exclusion of atypical or malignant features. Despite the low mortality rate associated with these cysts, prompt diagnosis and appropriate intervention are essential to reducing the potential for high morbidity. Recurrence remains a concern, necessitating personalized treatment strategies and, in some cases, consideration of more aggressive therapies upon recurrence. Given the unclear pathogenesis and need for more robust evidence, further data collection and research are crucial to developing standardized guidelines for diagnosing and managing peritoneal inclusion cysts.

## CRediT authorship contribution statement

All the authors participate in the treatment of the patients, writing, and approved the manuscript.

## Consent

Written informed consent was obtained from the patient for publication of this case report and accompanying images. A copy of the written consent is available for review by the Editor-in-Chief of this journal on request.

## Ethical approval

Not applicable.

## Guarantor

Mohamed Ali Chaouch.

## Research registration number

1. Name of the registry: N/A
2. Unique identifying number or registration ID: N/A
3. Hyperlink to your specific registration: N/A.

## Funding

This research received no specific grant from the public, commercial, or not-for-profit sectors.

## Patient consent

Written informed consent was obtained from the patient to publish this case report and accompanying images. On request, a copy of the written consent is available for review by the Editor-in-Chief of this journal.

## Declaration of competing interest

No conflict of interest to disclose.
